# Response by Tabák et al to Letters Regarding Article, “Risk of Macrovascular and Microvascular Disease in Diabetes Diagnosed Using Oral Glucose Tolerance Test With and Without Confirmation by Hemoglobin A1c: The Whitehall II Cohort Study”

**DOI:** 10.1161/CIRCULATIONAHA.123.063545

**Published:** 2023-03-21

**Authors:** Adam G. Tabák, Naveed Sattar, Mika Kivimäki

**Affiliations:** 1 UCL Brain Sciences, University College London, London, UK; 2 Department of Internal Medicine and Oncology, Semmelweis University Faculty of Medicine, Budapest, Hungary; 3 Department of Public Health, Semmelweis University Faculty of Medicine, Budapest, Hungary; 4 School of Cardiovascular and Metabolic Health, University of Glasgow, Glasgow, UK; 5 Clinicum, Faculty of Medicine, University of Helsinki, Helsinki, Finland

## In Response

We thank Schmidt, Deng and their colleagues for thoughtful comments on our paper.^1^ In agreement with other studies,^2^ our data from the Whitehall study show similarly higher risk of cardiovascular disease associated with incident diabetes at baseline based on a single measurement of HbA1c (RR: 1.66, 95%CI: 1.09-2.52) and oral glucose tolerance test (OGTT, 1.41, 95%CI: 1.04-1.90). Thus, we agree with the commentators that there is no inherent rank order between different glycaemic measures in predicting cardiovascular risk.

Rather than comparing glycaemic measures in cardiovascular disease prediction, the aim of our paper was to describe potential consequences of changing a glucose/OGTT-based diagnostic approach of diabetes mellitus to an HbA1c-based approach. We found that people whose OGTT-based diagnosis did not meet HbA1c criteria for diabetes during follow-up had a similar long-term cardiovascular disease risk as the background population. This finding is clinically important as it suggests that the current change from the OGTT-based to HbA1c-based diagnostic approach is not harming people, but rather appears beneficial. Unfortunately, the overlap of HbA1c measurements and OGTTs in our study is limited and thus we cannot answer the interesting question by Schmidt and colleagues regarding the risk associated with an HbA1c-diagnosed diabetes with or without OGTT-confirmation.

We agree with Schmidt et al that an unconfirmed diabetic glucose value may signal random (or non-random) variation and is not sufficient for diabetes diagnosis. However, there is sufficient evidence to show that the short-term repeatability of HbA1c values is substantially better than that of fasting or postload glucose.^3^ We confirmed this in an additional analysis of a subgroup of Whitehall participants with blood samples used to estimate methodological variability (the same sample was split and analysed twice) and biological variability (the test was repeated in the same individual within one month). We found that for HbA1c the correlation between the two values was similar in split samples and repeat samples (r=0.99, 95%CI: 0.98-0.995 vs r=0.97, 95%CI: 0.95-0.98), but for fasting glucose (r=0.98, 95%CI: 0.96-0.99 vs r=0.76 95%CI: 0.64-0.84) and for postload glucose (r=0.99, 95%CI: 0.98-0.99 vs. 0.68, 95%CI: 0.57-0.78) there was a substantially stronger correlation for the split samples than for repeat samples (unpublished data). In light of these observations, the observed ‘regression to the mean over 3.4 years’ in ELSA-Brasil may reflect both biological variability in glycaemic measures and the natural history of type 2 diabetes that includes frequent (up to 30%) natural remissions in people with newly diagnosed diabetes and an approximately 5% remission in those with a mean diabetes duration of 5 years.^4, 5^

Deng et al highlight that the association between vascular diseases and glycaemia is not limited to levels above the diabetes diagnostic values, though independent associations are clearer above HbA1c of 6.5%. However, there is only limited evidence suggesting that interventions to prevent or screen diabetes in high-risk individuals would reduce the risk of macrovascular disease in high-income countries with universal health care.^6^ Furthermore, to the best of our knowledge, it s not known what is the cut-off level of glycaemia associated with any ‘legacy effect’.

We agree with Deng et al that HbA1c has certain limitations as a diagnostic measure. However, compared to HbA1c, the other measures, such as the OGTT and continuous glucose monitoring based time in range, are either much more variable and complicated, or costly and labour intensive. Given this, their use is unlikely to be adopted for screening purposes.

Deng et al correctly pointed out that estimated glomerular filtration rate (eGFR) is not an ideal proxy for diabetic kidney disease (i.e., diabetic nephropathy). Unfortunately, urinary albumin, which is a better marker of diabetic nephropathy, was not measured in the Whitehall study. However, most chronic kidney disease cases (individuals with low eGFR) are due to diabetes mellitus and hypertension in high-income countries.^7^ To minimise other causes of chronic kidney disease, we performed a sensitivity analysis after the exclusion of persons with systemic autoimmune diseases and anaemia and the results remained unchanged.

Considering all the above points, our general conclusion is robustly supported by our findings. Even so, as the findings are from a single study, confirmation in other prospective cohort studies with diabetes diagnosis based on different glycaemic measures and long follow-up would be valuable.

## Disclosures

Prof Tabák is supported by the UK Medical Research Council (S011676) and the Ministry of Innovation and Technology of Hungary from the National Research, Development and Innovation Fund (2021 Thematic Excellence Programme funding scheme, TKP2021-NKTA-47). Prof Kivimäki is supported by the Wellcome Trust (221854/Z/20/Z), the Medical Research Council (R024227, S011676), the National Institute on Aging (R01AG056477, R01AG062553), and Academy of Finland (350426). Prof Sattar is supported by the British Heart Foundation Centre of Research Excellence Grant (RE/18/6/34217). Prof Sattar has consulted for Amgen, Astrazeneca, Boehringer Ingelheim, Eli-Lilly, Hanmi Pharmaceuticals, Novo Nordisk, Novartis, Roche Diagnostics, Sanofi, and Pfizer. Prof Sattar has also received grant funding, paid to his University, from Astrazeneca, Boehringer Ingelheim, Novartis and Roche Diagnostics.
